# Polyglycidol, Its Derivatives, and Polyglycidol-Containing Copolymers—Synthesis and Medical Applications

**DOI:** 10.3390/polym8060227

**Published:** 2016-06-09

**Authors:** Mateusz Gosecki, Mariusz Gadzinowski, Monika Gosecka, Teresa Basinska, Stanislaw Slomkowski

**Affiliations:** Center of Molecular and Macromolecular Studies, Polish Academy of Sciences, Sienkiewicza 112, 90-363 Lodz, Poland; gosecki@cbmm.lodz.pl (M.Gosecki); mariuszg@cbmm.lodz.pl (M.Ga.); mdybko@cbmm.lodz.pl (M.Gosecka); basinska@cbmm.lodz.pl (T.B.)

**Keywords:** polyglycidol, linear, branched, copolymer, nanoparticles, microparticles, carriers of bioactive compounds

## Abstract

Polyglycidol (or polyglycerol) is a biocompatible polymer with a main chain structure similar to that of poly(ethylene oxide) but with a –CH_2_OH reactive side group in every structural unit. The hydroxyl groups in polyglycidol not only increase the hydrophilicity of this polymer but also allow for its modification, leading to polymers with carboxyl, amine, and vinyl groups, as well as to polymers with bonded aliphatic chains, sugar moieties, and covalently immobilized bioactive compounds in particular proteins. The paper describes the current state of knowledge on the synthesis of polyglycidols with various topology (linear, branched, and star-like) and with various molar masses. We provide information on polyglycidol-rich surfaces with protein-repelling properties. We also describe methods for the synthesis of polyglycidol-containing copolymers and the preparation of nano- and microparticles that could be derived from these copolymers. The paper summarizes recent advances in the application of polyglycidol and polyglycidol-containing polymers as drug carriers, reagents for diagnostic systems, and elements of biosensors.

## 1. Introduction

Generally, polymers should be chemically stable to ensure the maintenance of their properties during storage, processing into products, and usage. Typical examples are polyethylene, polypropylene, and several other vinyl polymers. However, for many applications polymers with special properties resulting from the presence in their chains of properly selected reactive groups are needed, such as groups with hydroxyl, carboxyl, aldehyde, and amine functions.

Linear poly(ethylene oxide), one polymer that has found many applications in medicine in spite of some pros and contras, is sufficiently stable, hydrophilic, and biocompatible; when attached to surfaces it makes them protein repellent, and provides “stealth” properties to the poly(ethylene oxide)-modified nanoparticles [1,2,3,4,5,6]. However, the chains of linear poly(ethylene oxide) cannot have more than two reactive end-groups, whereas, in many instances, polymers with similar properties, but bearing multiple functional groups, are needed. Such multifunctional polymers are suitable for covalent immobilization of various drugs, receptor-targeting moieties, fluorescent labels, and many other uses.

**Scheme 1:**
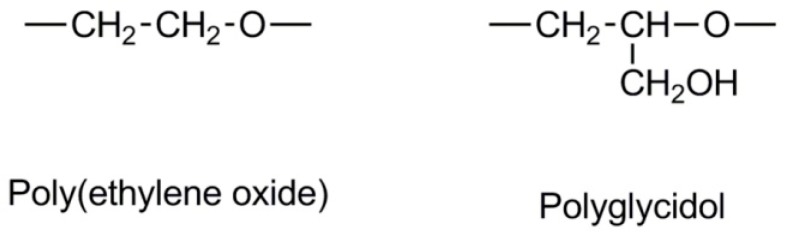
Poly(ethylene oxide) and polyglycidol constitutional units.

The closest multifunctional analogue of poly(ethylene oxide) is polyglycidol (see Scheme 1). The number of hydroxyl –CH_2_OH groups in the polyglycidol chain is equal to its degree of polymerization. Thus, polymers with high molar masses provide many options for immobilization and labeling.

Poly(ethylene oxide) and polyglycidol are used in medicine as protecting and stabilizing agents. Direct intravenous or oral administration of many bioactive drug components, particularly nucleic acids and almost all kinds of proteins, is ineffective. Various hydrolases and components of molecular and cellular immune system induce degradation and elimination of these species from the blood. Digestion accompanies oral administration of unprotected nucleic acids and proteins. Moreover, even if some large fragments of partially degraded proteins or nucleic acids administered orally succeed in crossing the endothelium barrier of the intestine and reaching the blood stream, the immune system would degrade them further and eliminate from the organism. Covalent binding of poly(ethylene oxide) to proteins and nucleic acids hinders their degradation by protecting them from enzymes, antibodies, and macrophages. Thus, modification of proteins, nucleic acids, and their carriers by immobilization of poly(ethylene oxide) significantly increases the circulation of these species in the blood [1,3,4,6,7,8,9]. This process is usually called pegylation (the term is derived from poly(ethylene glycol), denoting poly(ethylene oxide) with low molar mass).

In many instances, the protection of bioactive compounds by pegylation is insufficient. In some cases good results were obtained by using liposome or nanoparticle carriers with a protecting external layer rich in poly(ethylene oxide).

Dense grafting of poly(ethylene oxide) strongly increases the hydrophilicity of surfaces, making them resistant to protein adsorption and more biocompatible [2,10]. In the case of diagnostic devices, the tailored modification of surfaces with poly(ethylene oxide) reduces the uncontrolled, adventitious adsorption of proteins, which is important for the specificity of diagnostic tests [5,11].

Poly(ethylene oxide) is used for many medical applications. However, polyglycidol equipped with hydroxyl groups in each polymeric unit opens a way for many others.

There are thousands of original and review papers devoted to the medical applications of poly(ethylene oxide). On the contrary, the knowledge on medical applications of polyglycidol is significantly more limited. The aim of this paper is to give an overview of the synthesis of polyglycidol and polyglycidol-containing copolymers with varied microstructure and topology and some of their medical applications.

## 2. Synthesis of Polyglycidol- and Oilyglycidol-Containing Copolymers

### 2.1. Monomers

**Scheme 2:**
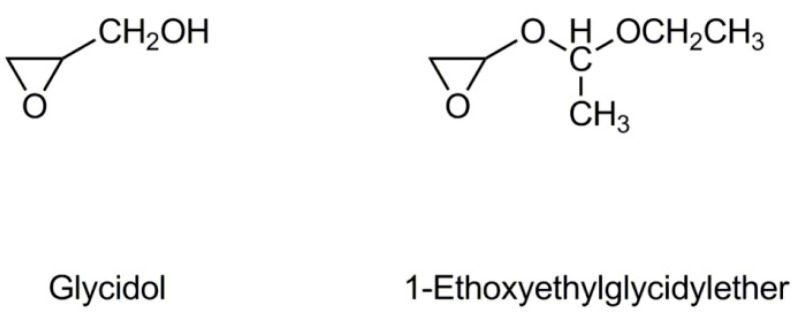
Structure of glycidol and 1-ethoxyethylglycidyl ether.

Glycidol contains two reactive groups in a molecule, epoxide and hydroxyl (Scheme 2). In anionic polymerization the epoxide group is involved in propagation, whereas the hydroxyl one is responsible for chain transfer reactions. Therefore, the polymerization of glycidol always leads to branched polymers. Linear polyglycidol could be synthesized only by polymerization of the monomer with blocked –CH_2_OH groups (e.g., with 1-ethoxyethyl or trityl moieties).

**Scheme 3:**
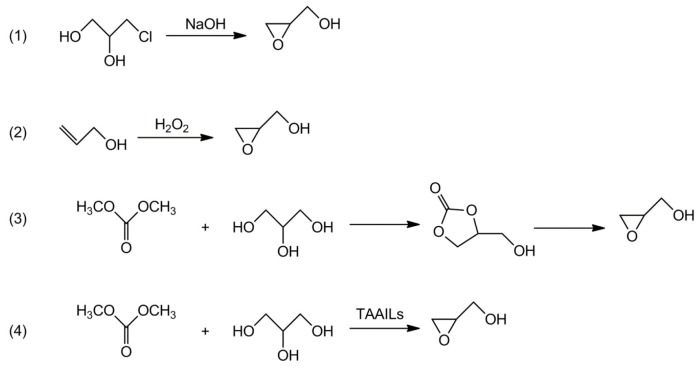
Synthetic routes to glycidol.

There are several routes for the synthesis of glycidol (see Scheme 3) [12,13,14,15,16,17,18,19,20,21]. Industrial production of glycidol is based on the epoxidation of allyl alcohol with hydrogen peroxide or on the conversion of glycerol into glycerol monochlorohydrin and its subsequent conversion into glycidol under the action of a strong base (e.g., KOH) [12,13]. However, these processes are not environmentally friendly, producing significant amounts of harmful waste and causing corrosion of equipment. Recently, some researchers concentrated their attention on the much more convenient approach based on decarboxylation of glycerol carbonate. Using tetraethylammonium amino acid ionic liquids (TAAILs) as catalysts allows us to carry out this synthesis in one pot [17,18,20,21].

**Scheme 4:**
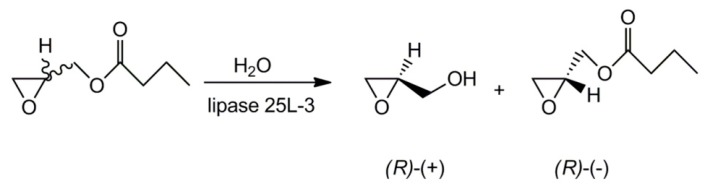
Synthetic pathway to enentiomerically pure glycidol.

The glycidol molecule contains a chiral carbon atom in the ring. Since for some applications the enantiomerically pure polyglycidol (or polyglycidol enriched in polymeric units of one enantiomer) might be needed, some researchers investigated the stereocontrolled synthesis of glycidol. Already in 1991, R. M. Hanson published an extensive review on this subject [12]. The reviewed methods included synthesis of the optically active monomers from the optically active substrates (e.g., from d-mannitol or l-serine), syntheses by asymmetric epoxidation, enzymatic transformation of achiral substrates, and chiral resolution. The latter is especially interesting because it could be based on the already synthesized glycidol with blocked –CH_2_OH groups. Using the properly chosen lipase and hydrolysis conditions allowed synthesis of glycidol with nearly 100% enantiomeric purity (see Scheme 4) [22].

**Scheme 5:**
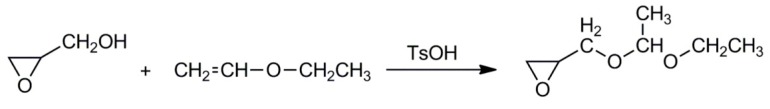
Synthetic route to 1-ethoxyethylglycidyl ether.

Synthesis of linear polyglycidol requires a monomer with blocked –CH_2_OH groups. Otherwise, the unblocked groups participate in chain transfer, which leads to the branching. Fitton *et al.* described synthesis of glycidol in which the hydroxyls were blocked with 1-ethoxyethyl groups [23]. The blocked monomer was synthesized in a simple reaction of glycidol with vinyl ethyl ether. The process was catalyzed with *p*-toluenesulfonic acid (see Scheme 5).

### 2.2. Synthesis of Linear Polyglycidol

In 1994 N. Spassky and co-workers provided the first reliable data on the synthesis of linear polyglicidol with molar masses up to 30,000 [24]. The polymer was obtained by anionic polymerization of 1-ethoxyethylglycidyl ether (blocked glycidol) initiated with cesium hydroxide and by deblocking removing protecting groups at acidic conditions.

**Scheme 6:**
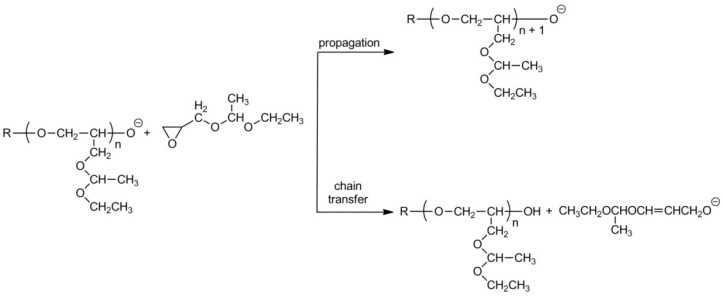
Propagation and cain transfer in the anionic polymerization of 1-ethoxyethylglycidyl ether.

Mono- and difunctional potassium alkoxides and sec-BuLi/phosphazene base were used later as initiators [25,26,27,28,29,30]. However, detailed studies of the anionic polymerization of 1-ethoxyethylglycidyl ether revealed that in these processes propagation is accompanied by chain transfer, which leads to polymers with decreased molar masses (see Scheme 6 [28]).

**Scheme 7:**
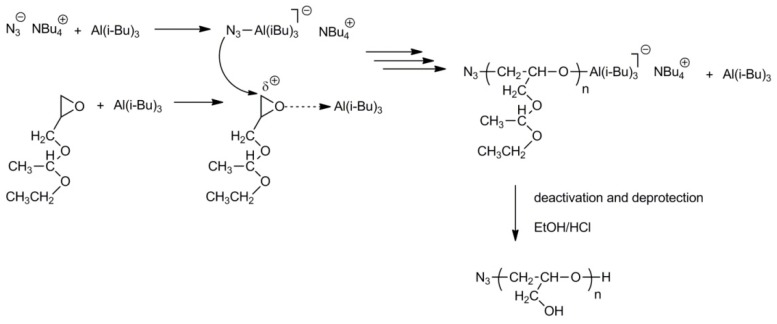
Polymerization of 1-ethoxyethylglycidyl ether with tetrabutylammonium azide initiator and triisobutylaluminum catalyst (monomer activator).

Polymers with higher molar masses (up to 85,000 for poly(1-ethoxyethylglycidyl ether) and 53,000 for poly(*tert*-butyl glycidyl ether)) were obtained by monomer-activated anionic polymerization initiated with tetraoctylammonium bromide or tetrabutylammonium azide initiators and a triisobutylaluminum catalyst [29,31]. Deblocking of hydroxyl groups in the abovementioned polymers gave polyglycidol samples with *M*_n_ = 74,000 and 30,200, respectively. The mechanism of initiation and propagation for the polymerization of 1-ethoxyethylglycidyl ether in a process with tetrabutylammonium azide initiator and triisobutylaluminum catalyst (monomer activator) is shown in Scheme 7.

Activation of the monomer molecule with Al(*i*-Bu)_3_ leads to formation of a partial positive charge at the endocyclic –CH_2_– group, which facilitates the nucleophilic attack during the initiation and propagation steps.

Polymerization of 1-ethoxyethylglycidyl ether and deblocking of –OH groups is not the only pathway to creating linear polyglycidol. Other monomers, like trimethylsilylglycidyl ether and *t*-butylglycidyl ether, could also be used for this purpose [32]. It is worth noting that *t*-butylglycidyl ether is commercially available. However, the advantage of synthesis via polymerization of 1-ethoxyethylglycidyl ether consists is the easy deblocking of –OH groups by removal of acetal moieties in mildly acidic conditions.

In some instances the difference in deprotection rates for acetal and *t*-butyl groups could be beneficial. For example, in Section 2.7 (on functionalized polyglycidol and polyglycidol-containing copolymers) we discuss the synthesis of polyglycidol derivatives with different labels, based on the selective removal of acetal and *t*-butyl groups in poly(1-ethoxyethylglycidyl ether)-*b*-poly(*t*-butylglycidyl ether).

### 2.3. Synthesis of Star-Like Polyglycidol

Polymerization of blocked glycidol with multifunctional initiators leads to star-like polymers, which after deblocking yield star-like polyglycidols [26,33].

**Scheme 8:**
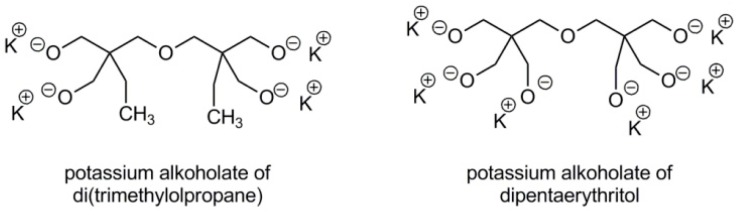
Multifunctional initiators used for polymerization of blocked glycidol.

Examples of multifunctional initiators are shown in Scheme 8.

The number of arms in the star-like polyglycidol is equal to the number of hydroxyl groups in the alcohol used for synthesis of the initiator.

### 2.4. Synthesis of Branched Polyglycidol

**Scheme 9:**
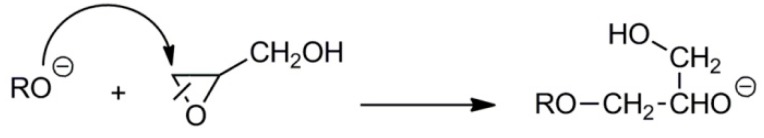
Monomer addition in the anionic polymerization of glycidol.

In anionic polymerization of glycidol, the propagation step consists of the nucleophilic attack of the alkoxide active center on the endocyclic CH_2_ group and the scission of the CH_2_–O bond. As a result, the newly added unit contains the primary hydroxyl side group (see Scheme 9).

**Scheme 10:**
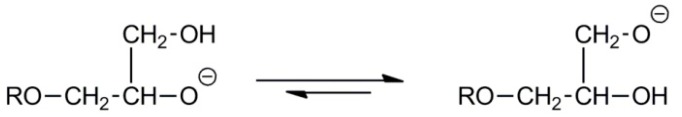
Intramolecular chain transfer in the anionic polymerization of glycidol.

However, the intramolecular chain transfer leads to formation of the primary alkoxide propagating species (see Scheme 10) [34].

**Scheme 11:**
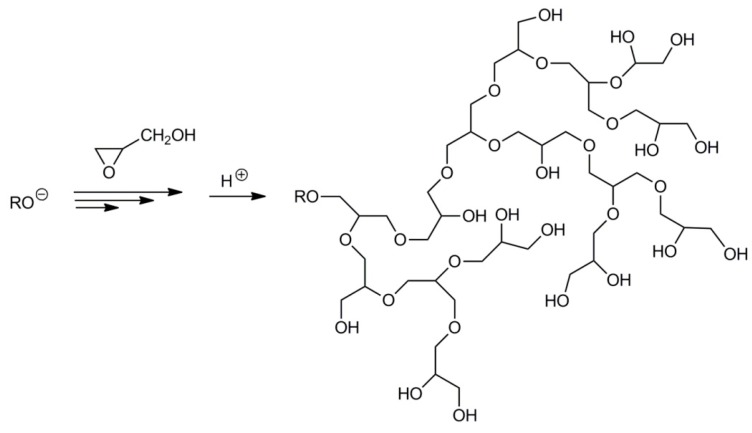
Hyperbranched polymer formed in the anionic polymerization of glycidol.

Because in polymer chains the primary and secondary hydroxyl side groups can be converted into propagating alkoxide species, branching is inevitable (see Scheme 11) [34]. It is worth noting that not all hydroxyl groups participate in the chain transfer leading to branching. Thus, after completion of the polymerization process and the killing of the active species, the branched macromolecules may contain primary and secondary hydroxyl groups not only at the chain ends but also along the chains, inside the branches.

It has been established that the slow addition of glycidol allows for obtaining branched polymers with a narrow molar mass distribution [35,36,37]. The following considerations explain these observations.

Alkoxides of primary and secondary alcohols differ with respect to their reactivity. Thus, a narrow molar mass distribution is possible only when at any time in each growing macromolecule the primary and secondary propagating species are present with the same probability, *i.e.*, when for each growing chain the apparent rate of propagation is the same. Every addition of a monomer molecule leads to formation of the propagating specie containing an alkoxide of secondary alcohol and a primary hydroxyl side group. Thus, to maintain the same probability of –CH_2_O^−^ and =CHO^−^ in every macromolecule, the average time between the addition of monomer molecules should be longer than the time needed for establishing equilibrium between the abovementioned alkoxides. Such conditions could be assured by slow monomer additions, maintaining low monomer concentration during the whole polymerization process.

**Scheme 12:**
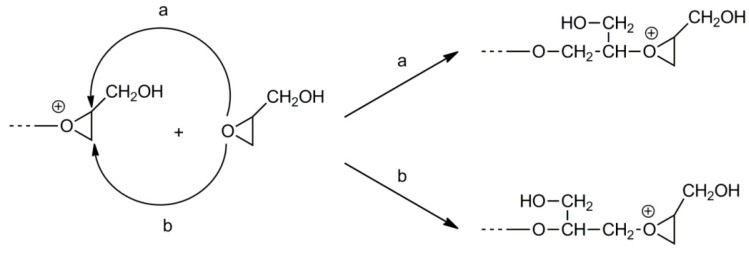
Monomer addition routes in the cationic polymerization of glycidol.

Glycidol also polymerizes by cationic means. Cationic polymerization of oxiranes proceeds according to the active chain-end (ACE) and/or activated monomer (AM) mechanisms [38]. In polymerization proceeding by ACE mechanism, the active species are tertiary oxonium ions located at the ends of growing chains. For glycidol, the propagation by the ACE mechanism, independently of the ring-opening mode during propagation, leads exclusively to polymers with primary side hydroxyl groups (see Scheme 12).

Propagation according to the AM mechanism consists of a reaction of protonated monomer molecules with the hydroxyl end-groups of the growing chains. Depending on the mode of ring scission in the activated monomer molecule, the primary and secondary hydroxyl groups are formed.

A study by S. Penczek *et al.* revealed that in the cationic polymerization of glycidol initiated with Brønsted acids the AM mechanism plays an important role [38]. Because activated glycidol also reacts with hydroxyl groups along the polyglycidol chains, branching is inevitable.

**Scheme 13:**
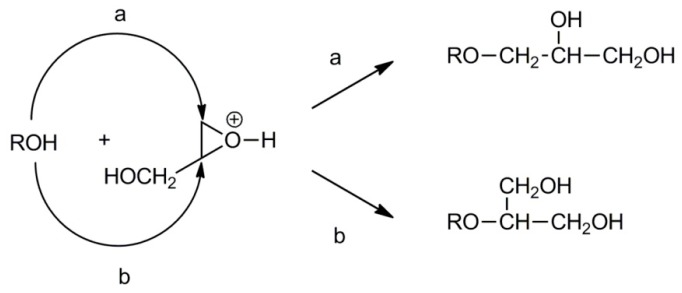
Cationic polymerization of glycidol by the activated monomer mechanism.

The polymer formed after the complete monomer conversion contains the primary and secondary hydroxyl groups. The primary hydroxyl groups are the exocyclic groups remaining from the monomer. The secondary hydroxyl groups are formed as a result of propagation along the pathway, as shown in Scheme 13.

### 2.5. Synthesis of Polyglycidol with Varied Topology by Grafting from the Linear Polyglycidol

**Scheme 14:**
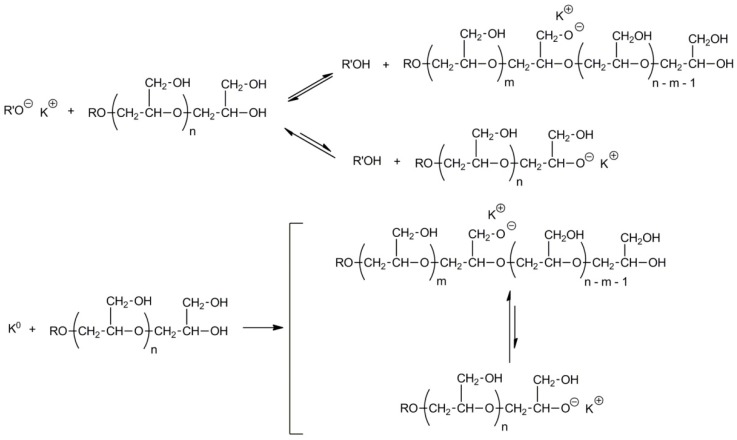
Macroinitiators on the basis of linear polyglycidol.

Linear polyglycidol containing hydroxyls not only as the end groups but also along the chain could be functionalized and modified in various ways. Recently Frey discussed this subject in a detailed manner [32]. Equilibration of low molar mass metal alkoxides with polyglycidol converts some hydroxyl groups along the polymer chain into alkoxides, which are able to initiate the polymerization of oxiranes and cyclic esters (see Scheme 14). Alkoxide anions along the polyglycidol chain could also be formed by contact of the polyglycidol solution with sodium or a potassium mirror. Since reactions of alkoxide and hydroxyl groups are reversible, a new chain may be grown from practically every hydroxyl group.

**Scheme 15:**
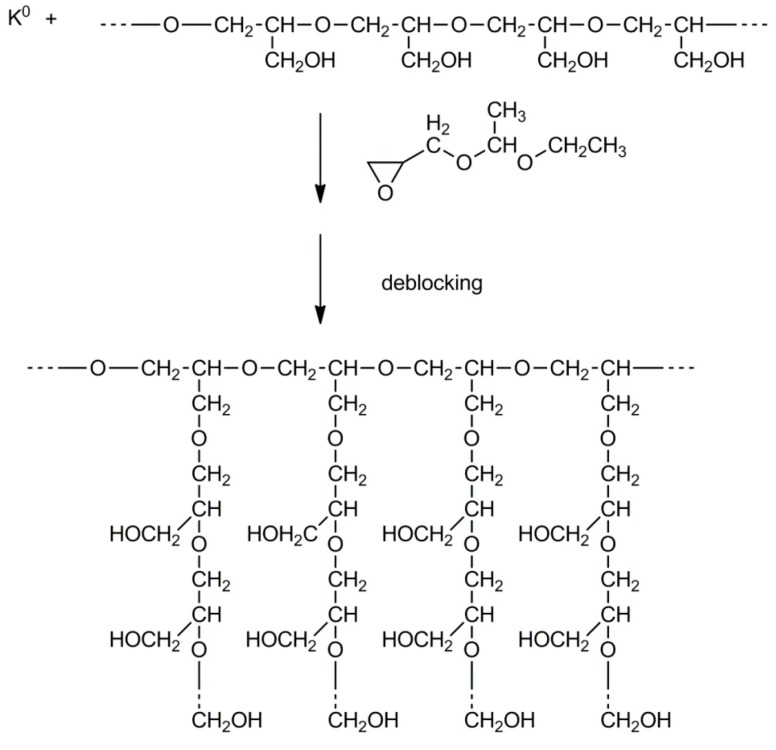
Synthesis of comb-like polyglycidol.

**Scheme 16:**
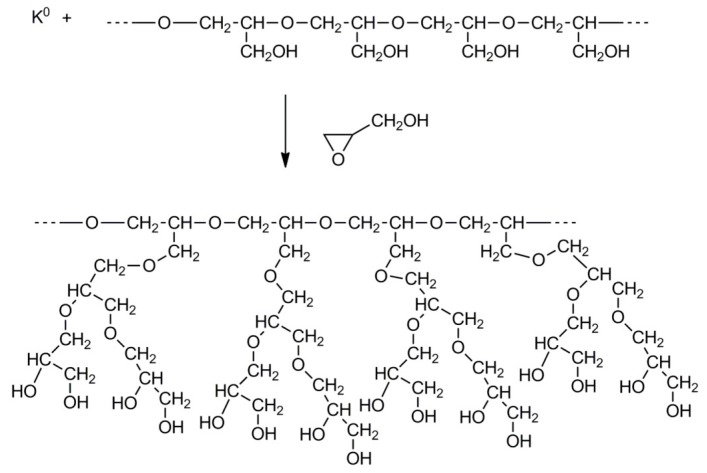
Linear polyglycidol with hyperbranched grafts.

The structure of the obtained polymer depends on the monomer used for the synthesis of the grafts. For example, formation of grafts from linear polyglycidol carried out by polymerization of blocked glycidol (e.g., 1-ethoxyethylglycidyl or *tert*-butylglycidyl ether) yields polymers with a comb-like structure, whereas grafting of glycidol would result in hyperbranched grafts. The abovementioned processes are shown in Scheme 15 and Scheme 16, illustrating syntheses of polyglycidol with linear and hyperbranched grafts, respectively.

### 2.6. Synthesis of Polyglycidol-Containing Copolymers

The presence of hydroxyl groups (which may be protected or not) in glycidol and glycidol-related monomers opens possibilities for the synthesis of a variety of polyglycidol-containing copolymers. Generally, the possible routes include:

- synthesis of the linear di-block copolymers by polymerization of glycidol with blocked hydroxyl groups in the process initiated with macroinitiator, *i.e.*, with other polymers containing active centers at the ends of their chains;
- synthesis of the linear di-block copolymers using polyglycidol with blocked hydroxyl groups along the chain and active end groups, as an initiator for the polymerization of other comonomers;
- synthesis of the linear tri-block copolymers using di-block copolymers with active end groups (obtained by routes a or b) as macroinitiatiors for the polymerization leading to addition of the third block;
- synthesis of the linear tri-block copolymer by polymerization of glycidol with a blocked hydroxyl group, initiated by a di-functional initiator and using the synthesized di-block macroinitiator for the polymerization of other monomers, resulting in the addition of new blocks at both ends;
- synthesis of the linear tri-block copolymer in a way similar to route d but by using glycidol with blocked hydroxyl groups as a second comonomer;
- synthesis of a comb-like copolymer by using polyglycidol (with unprotected hydroxyl groups) as a macroinitiator and catalyst (e.g., Sn(Oct)_2_) for polymerization of other comonomers;
- synthesis of the branched copolymer in a way similar to route f but using hyperbranched polyglycidol as a macroinitiator;
- synthesis of the linear-hyperbranched copolymer in a way similar to route a but using glycidol (*i.e.*, a monomer with unblocked hydroxyl groups) as a second monomer;
- synthesis of the hyperbranched copolymer by polymerization of glycidol from reactive groups in the corona of another hyperbranched copolymer.

**Scheme 17:**
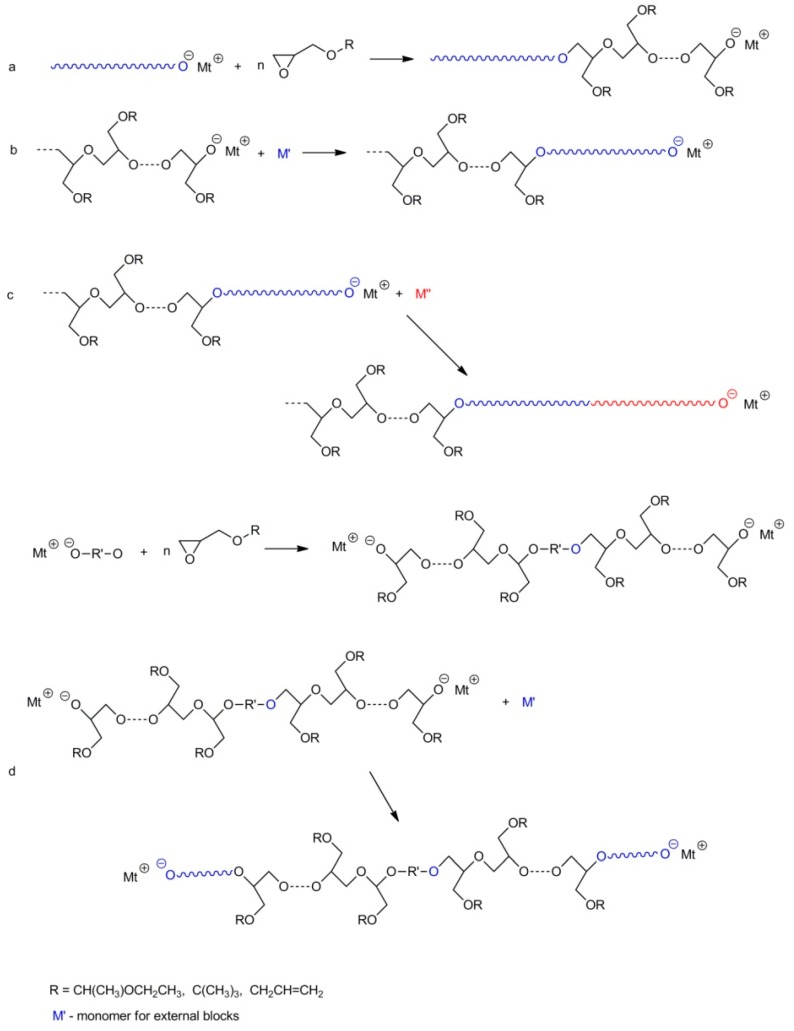
Selected routes leading to copolymers with blocked polyglycidol blocks.

**Scheme 18:**
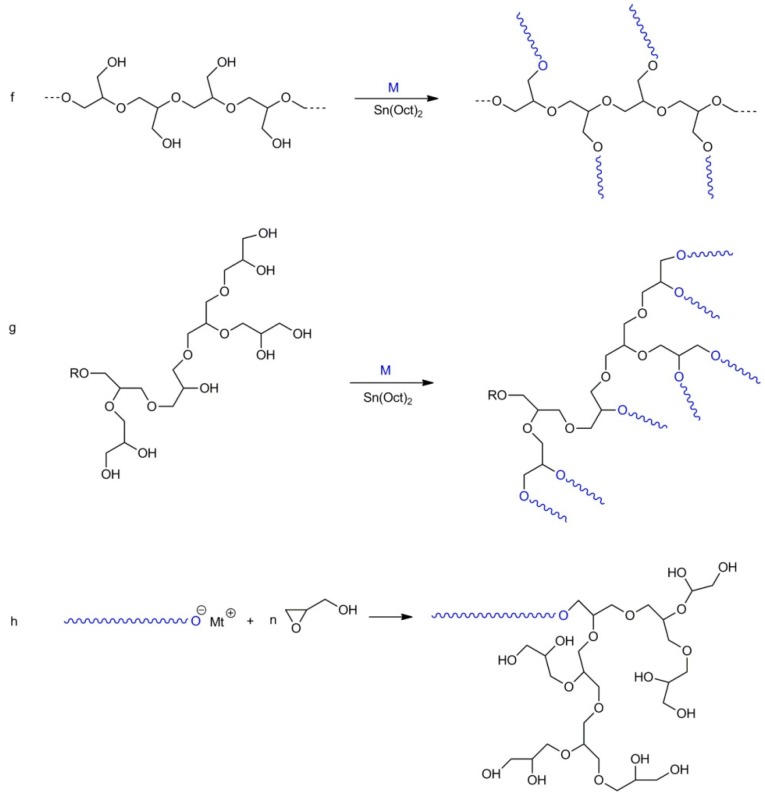
Selected routes leading to copolymers with blocked polyglycidol blocks.

Scheme 17 and Scheme 18 illustrate some of the abovementioned routes.

Not all routes shown in Scheme 17 and Scheme 18 were verified experimentally. However, taking into account our present knowledge, they all should allow for synthesis of the desired products. Below are listed some examples of copolymer synthesis.

**Scheme 19:**
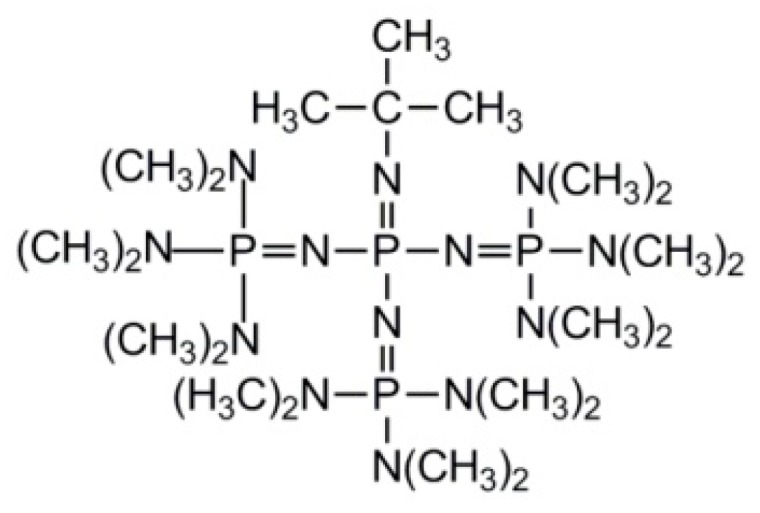
Structure of the P(4)-*t*-Bu phosphazene base.

Möller *et al.* synthesized polystyrene-*b*-polyglycidol in a sequential polymerization of styrene and 1-ethoxyethylglycidyl ether (blocked glycidol) with subsequent deprotection of hydroxyl groups, using concentrated HCl or acidic ion-exchange resins [39,40]. It should be mentioned that polystyrene and polydiene blocks are usually obtained using lithium initiators. This is especially important in the case of dienes like butadiene and isoprene, when a high content of 1–4 units is required. Polystyrene–Li and polydiene–Li active species are sufficiently reactive to add epoxide molecules. However, lithium alkoxides formed as a result of the abovementioned additions are much less reactive and are unable to add subsequent monomer molecules [41,42]. As a result, the one-pot chain extension of polyvinyl block with poly(1-ethoxyethylglycidyl ether) is ineffective. Thus, before continuation of the synthesis the exchange of Li^+^ for K^+^ or Cs^+^ cations is necessary, making the synthesis more tedious. The problem was solved when the polymerization of epoxides promoted by phosphazene base P(4)-*t*-Bu (see Scheme 19) strongly complexing Li^+^ was developed [43,44].

Addition of P(4)-*t*-Bu at the beginning of the synthesis of the second block (*i.e.*, at the beginning of the polymerization of epoxides) results in the complexation of Li^+^ cations. Formation of a Li^+^/P(4)-*t*-Bu complex shifts the equilibrium between the covalent lithium alkoxides and the much more reactive ionic species towards the latter ones. This approach allowed for obtaining polystyrene-*b*-polyglycidol copolymers with degrees of polymerization ranging from 40 to 160 and from 70 to 220 for polystyrene and polyglycidol blocks, respectively [40].

Macroinitiators obtained in the reaction of poly(styrene-*co*-butadiene-*co*-isoprene)–OH with potassium hydride were used for the synthesis of copolymers containing polyvinyl and poly(1-ethoxyethylglycidyl ether) blocks. The successful deprotection with concentrated HCl yielded copolymers with polyglycidol blocks [45].

**Scheme 20:**
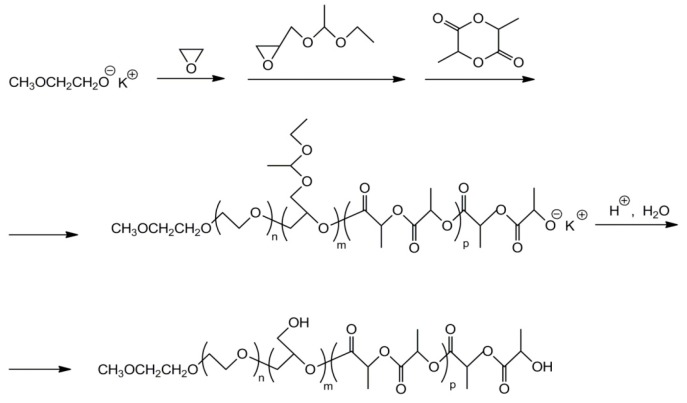
Synthesis of tri-block copolymer with poly(ethylene oxide), polyglycidol and polylactide blocks.

Especially interesting are copolymers containing the hydrophobic biodegradable polyester and the hydrophilic polyglycidol blocks. These copolymers are considered candidates for the preparation of drug carriers. To this class belong poly(ethylene oxide)-*b*-polyglycidol-*b*-poly(l-lactide) tri-block copolymers, which were synthesized by a sequential polymerization of ethylene oxide, 1-ethoxyethylglycidyl ether and l-lactide and by deprotection converting acetal groups in poly(1-ethoxyethylglycidyl ether) into hydroxyl groups (Scheme 20) [46].

A linear tri-block copolymer with central polyglycidol and side polylactide blocks was synthesized by one-pot polymerization of 1-ethoxyethylglycidyl ether initiated with HOC(CH_3_)_2_CH_2_CH_2_C(CH_3_)_2_O^−^K^+^ (a derivative of 2,4-dimethylhexane-2,4-diol), with subsequent (when the first monomer was consumed) polymerization of l-lactide [30]. Due to the proton exchange, l-lactide polymerized at both ends. Deprotection of hydroxyl groups, carried out using AlCl_3_∙6H_2_O, was highly selective. Only the acetal groups were cleaved, leaving the ester groups in the poly(l-lactide) blocks intact.

It should be noted that in the synthesis of linear copolymers with polyglycidol and polylactide blocks the sequence of block synthesis is important. At least for K^+^ counterions, the poly(1-ethoxyethylglycidyl ether) block should be synthesized first. This is because the …-CH[CH_2_OCH(CH_3_)OCH_2_CH_3_]O^−^K^+^ growing species, located at the ends of living poly(1-ethoxyethylglycidyl ether), initiate the polymerization of l-lactide, whereas …-C(O)CH(CH_3_)O-K^+^ species of living polylactide are inefficient as initiators of the polymerization of 1-ethoxyethylglycidyl ether. However, such a limitation does not apply to the copolymerization of *tert*-butyl glycidyl eter with ε-caprolactione, initiated with benzyl alcohol/ P(4)-*t*-Bu system, for which it was possible to obtain random copolymers [47]. Molar masses of synthesized copolymers were in the range of 13,000 to 49,200. Hydrolysis of *t*-Bu–O–CH_2_– groups in copolymers yielded polyglycidol units.

Pluronic-type copolymers in which polyglycidol constituted the external blocks were also synthesized [48]. The synthesis began with activation of both ends of the hydroxyl-terminated poly(propylene oxide) with CsOH·H_2_O. Water produced at this stage was removed by the addition of dry benzene and azeotropic distillation, yielding a poly(propylene oxide) di-functional macroinitiator containing Cs^+^ alcoholate active enters. The macroinitiator initiated polymerization of 1-ethoxyethylglycidyl ether and the tri-block copolymer—poly(1-ethoxyethylglycidyl ether)-*b*-poly(propylene oxide)-*b*-poly(1-ethoxyethylglycidyl ether) was obtained. Acetal groups in poly(1-ethoxyethylglycidyl ether) were cleaved with AlCl_3_∙6H_2_O, yielding the polyglycidol-*b*-poly(propylene oxide)-*b*-polyglycidol tri-block copolymer.

There are reports on the synthesis of polyglycidol-containing graft copolymers with branched polyglycidol grafts or with branched polyglycidol cores [49,50].

**Scheme 21:**
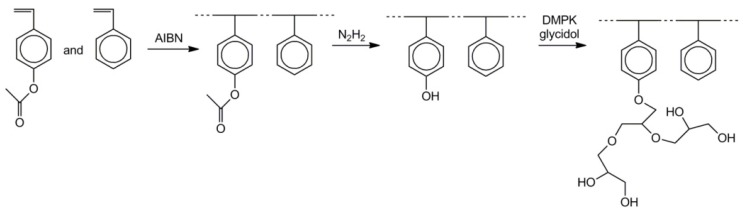
Synthesis of polystyrene with branched polyglycidol grafts.

A polystyrene main chain was synthesized by RAFT copolymerization of styrene and 4-acetoxystyrene initiated by *S*-1-dodecyl-*S’’*-(α,α’-dimethyl-α’’-acetic acid) trithiocarbonate as a chain-transfer agent [49]. Hydrolysis of acetoxy groups yielded poly(*p*-hydroxystyrene) units. The activation of hydroxyl groups with diphenylmethyl potassium (DPMK) yielded a macroinitiator initiating polymerization of glycidol. At this step the branched polyglycidol grafts were formed. The process is illustrated in Scheme 21.

The branched polyglycidol core of copolymer with poly(l-lactide) multi-arms was synthesized by ring-opening polymerization of glycidol catalyzed/initiated with Sn(Oct)_2_. The addition of d-lactide after complete conversion of glycidol leads to formation of the external poly(l-lactide) arms [50]. This copolymerization conforms to route g in Scheme 18, with M denoting d-lactide.

### 2.7. Functionalized Polyglycidol and Polyglycidol-Containing Copolymers

Functionalized polyglycidol could be obtained by the introduction of required functional groups during initiation, termination, or as a result of reactions with polyglycidol –CH_2_OH side groups.

For example, the synthesis of branched polyglycidol with primary amine groups was performed in a process that consisted of the following steps [51]:

- initiation of the polymerization of ethylene oxide with cesium dibenzyl-2-aminoethanolate;
- using obtained macroinitiator for initiation of the polymerization of 1-ethoxyethylglycidyl ether;
- deblocking of hydroxyl groups in poly(1-ethoxyethylglycidyl ether), activation of hydroxyl groups with CsOH;
- polymerization of glycidol initiated with …–CH_2_O^−^Cs^+^;
- conversion of the dibenzyl-2-aminoethyl-O–… groups into the NH_2_CH_2_CH_2_O–… groups.

**Scheme 22:**
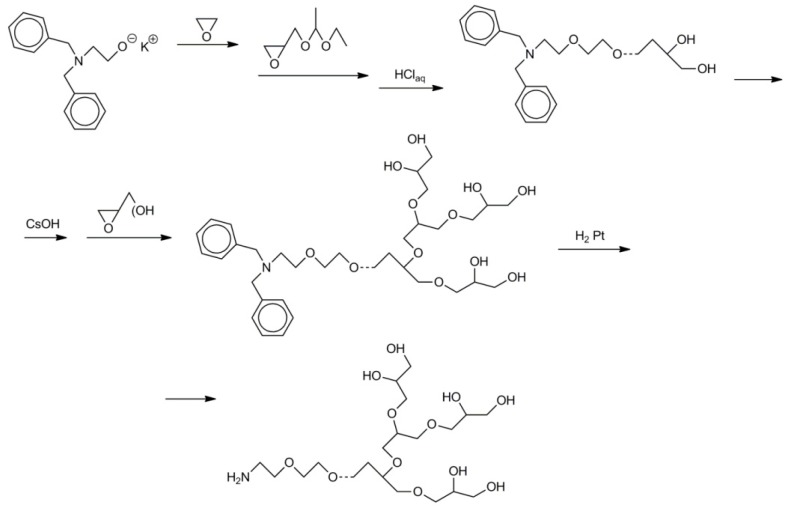
Synthesis of branched polyglycidol with primary amine groups.

Details of the synthesis are shown in Scheme 22.

It was also possible to obtain the sulfonate derivative of the abovementioned polymer [51].

The amine groups in branched polyglycidol, shown in Scheme 22, were further modified by reaction with 1-pyrene-butyric acid pentafluorophenyl ester. The reaction resulted in the addition of the pyrene moiety. The pyrene labels promoted adsorption of the strongly hydrophilic, branched polyglycidol onto the multi-walled carbon nanotubes, ensuring their solubilization with water [51].

End-capping of the living poly(1-ethoxyethylglycidyl ether) with *p*-chloromethyl styrene yielded α-*t*-butoxy-ω-vinylbenzyl-poly(1-ethoxyethylglycidyl ether), which after deprotection with formic acid gave α-*t*-butoxy-ω-vinylbenzyl-polyglycidol [52]. The macromonomer was used as a surfmer, stabilizing microspheres synthesized in the radical emulsion polymerization of styrene [53].

**Scheme 23:**
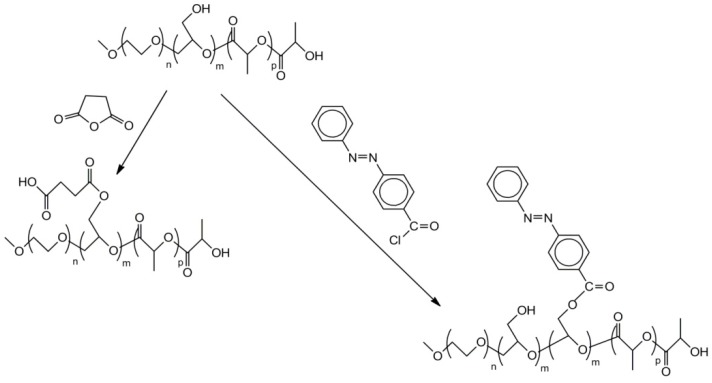
Synthesis of tri-block copolymer with poly(ethylene oxide), polyglycidol and polylactide blocks with carboxylic acid and 4-(phenyl-azo-phenyl) groups.

Functionalization of polyglycidol blocks in the tri-block copolymers (see Scheme 20) was conveniently performed by esterification of its hydroxymethyl groups with acid chlorides or with cyclic anhydrides [54]. These functionalization processes are illustrated in Scheme 23.

In 2010 Möller developed an elegant route for the synthesis of polyglycidol with some blocks labeled with side C_12_, C_14_, and C_16_ alkyl chains [55]. The route was based on observation that in acidic conditions the deblocking of hydroxyl groups in poly(*t*-butylglycidyl ether)-*b*-poly(1-ethoxyethylglycidyl ether) does not proceed at the same rate. Hydrolysis of acetal groups is much easier than hydrolysis of *t*-butyl ether groups. However, it is worth noting that selective deprotection of hydroxyl groups in random copolymers of *t*-butylglycidyl ether and 1-ethoxyethylglycidyl ether was not successful [56]. Apparently, the neighboring group effect facilitated the hydrolysis of *t*-butyl ether groups adjacent to the solvated hydroxyls, which were formed by fast hydrolysis of acetal groups [56]. Thus, the synthesis of polyglycidol, in which the units labeled with alkyl chains constituted a separate block, was performed by the following sequence of reactions:

- sequential polymerization of 1-ethoxyethylglycidyl ether and *t*-butylglycidyl ether initiated with C_6_H_5_–(CH_2_)_3_O^−^K^+^;
- deblocking of hydroxyl groups in poly(1-ethoxyethylglycidol ether) with hydrochloric acid (the concentration of HCl and time of deblocking were optimized);
- labeling of polyglycidol with alkyl chains in reaction between –CH_2_OH groups of polyglycidol and alkyl chains with isocyanate end-groups;
- deprotection of hydroxyl groups in poly(*t*-butylglycidyl ether) block using trifluoroacetic acid.

**Scheme 24:**
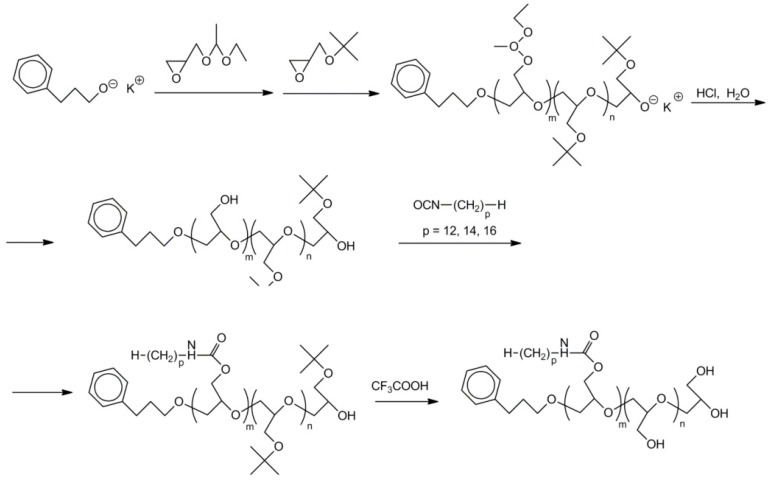
Synthesis fof polyglycidol with C_12_, C_14_, and C_16_ alkyl side chains.

The process of synthesis is illustrated in Scheme 24.

**Scheme 25:**
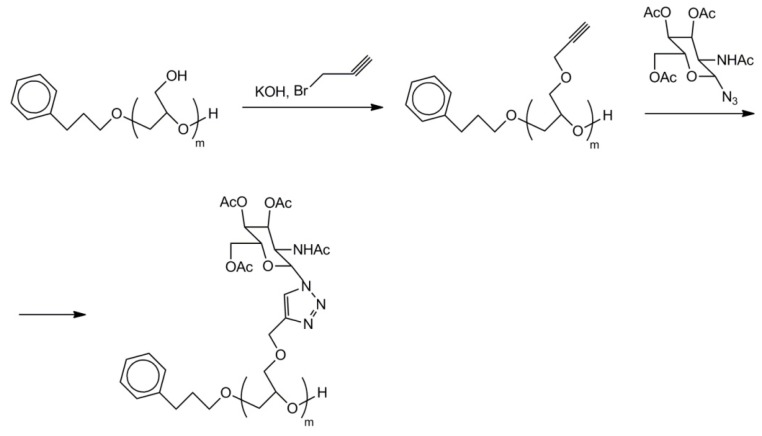
Polyglycidol labeled with sugar moieties.

Polyglycidol and polyglycidol-containing copolymers could also be conveniently functionalized by click reaction [56]. The process consists of the addition of propargyl moieties in a reaction of propargyl bromide with KOH-activated –CH_2_OH groups of polyglycidol. The obtained poly(glycidyl propargyl ether-*co*-glycidol) is ready for further functionalization by Cu(I) controlled (2 + 3) cycloaddition of compounds with azide groups. Scheme 25 gives an example of the addition of a sugar moiety.

The above-described modifications of polyglycidol and polyglycidol-containing copolymers yielded a variety of amphiphilic polymers, which, depending on the details of the structure of the hydrophilic and hydrophobic components, are often able to self-organize into nanoparticles, liposomes, and other polymer aggregates suitable for encapsulation or solubilization of hydrophobic compounds. The presence of reactive groups in polyglycidol and polyglycidol-functionalized derivatives allows their labeling with fluorescent labels [51,54].

The functional derivatives of polyglycidol can be used as building blocks for the preparation of nano-objects useful for various applications in medicine.

## 3. Applications of Polyglycidol and Polyglycidol-Containing Polymers in Medicine

### 3.1. Biocompatibility

Biocompatibility of polymers is indispensable for using these materials in medicine as drug carriers, implants (regardless whether permanent or resorbable), and various elements of equipment. Thus, the issue of the biocompatibility of polyglycidol is of primary importance.

**Scheme 26:**
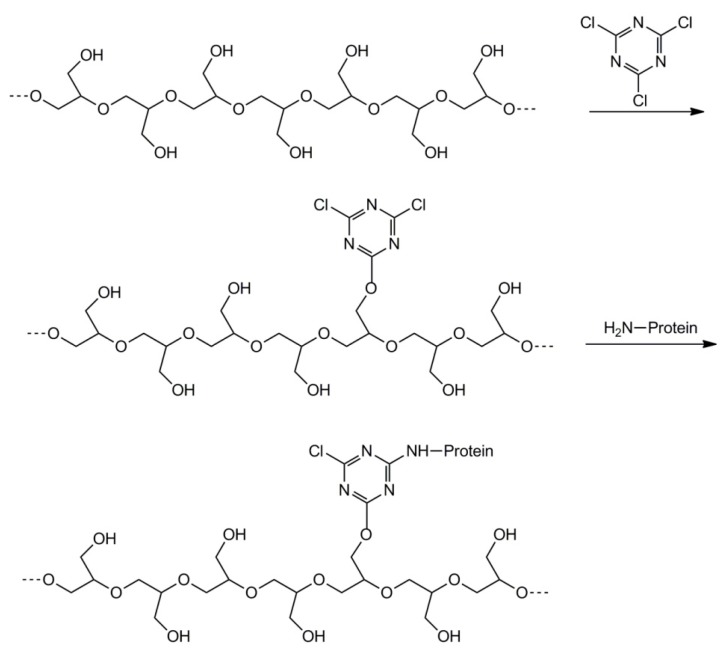
Attachment of protein to polyglycidol.

Efficient protein adsorption, accompanied by conformational changes resulting in the denaturation of adsorbed proteins, usually triggers undesired effects including immune response and clot formation, and has a deleterious influence on the biological functions of adsorbed proteins. Extensive studies carried out in our laboratory revealed that the incorporation of polyglycidol into the interfacial layer of the polystyrene microspheres and surfaces of carbon glass, gold, and stainless steel flat substrates leads to a strong reduction of protein adsorption [57,58,59,60,61,62]. However, the activation of polyglycidol hydroxyl groups with 1,3,5-trichlorotriazine allows for efficient covalent protein binding (see Scheme 26) [57,59,61,62].

Studies of interactions of linear and branched polyglycidols and polyglycidol-containing copolymers with cell cultures and living organisms revealed that these compounds:

- do not interact with the DNA of the Chinese hamster B14 cell line (linear and arborescent polymers with poly(ethylene oxide) and polyglycidol units) [63];
- are not toxic towards peripherial blood mononuclear cells (hyperbranched) [64];
- are hemocompatible (hyperbranched polyglycidol and hyperbranched copolymer of glycerol and sebacic acid) [65,66];
- and are well tolerated even if injected in high doses [65].

It was also found that dendritic polyglycidol with terminal sulfate end groups has anti-inflammatory properties [67]. These observations support the attempts to use polyglycidols with various architectures as well as their copolymers as materials for medical applications.

### 3.2. Polyglycidol-Based Drug Carriers

Many papers on drug carriers based on polyglycidol and polyglycidol derivatives have already been published. A comprehensive discussion of particular systems exceeds the scope of this paper. We will only present a list providing brief information on encapsulated bioactive compounds, the chemical composition of nano- or micro-carriers, and methods used for their preparation. The data are listed in Table 1.

### 3.3. Applications of Polyglycidol and Polyglycidol Derivatives in Diagnostics-Based Drug Carriers

Biocompatibility, long circulation time in the blood, the availability of many routes for functionalization, and the suitability for controlled binding of various biomolecules makes polyglycidol and its derivatives interesting for applications in medical diagnostics, both *in vivo* and *in vitro*.

There is great interest in contrast agents used in tissue imaging. Gd^3+^-loaded hyperbranched polyglycidol with amine groups containing corona [103] and Fe_3_O_4_ superparamagnetic particles protected by a hyperbranched pololyglycidol coating, used to stabilize particle circulation in the blood, were found to be suitable for Magnetic Resonance Imaging (MRI) [104,105]. Hyperbranched polyglycidol with sulfated corona, labeled with indocyanine green or indotricarbocyanine dyes, were excellent agents for near-infrared fluorescence imaging [106,107].

Complex particles comprising a superparamagnetic iron oxide core coated with fluorescent silica and protected with grafted hyperbranched polyglycidol constitute a new contrast agent for MRI and optical imaging [108]. Tri-modal imaging systems were also developed based on hyperbranched polyglycidol labeled with radioactive ^111^In, fluorescent Alexa dye, and Gd^3+^ for MRI [109]. Hyperbranched polyglycidol with moieties chelating ^67^Ga/^68^Ga were studied as reagents for positron emission tomography (PET) [110]. Gold nanorods coated with hyperbranched polyglycidol with sulfated corona were used as a contrast agent for optoacoustic tomography, for the monitoring of rheumatoid arthritis [111].

Polyglycidol with immobilized glucose oxidase is an essential component of biosensors for the detection of glucose [112].

Basinska *et al.* developed a new type of diagnostic test based on the changes of electrophoretic mobility in poly(styrene/α-tertbutoxy-ω-vinylbenzyl-polyglycidol) [113]. The test was dedicated for determination of γ-globulins against *Helicobacter pylori* in the blood serum. The microspheres were synthesized by emulsion copolymerization of styrene and α-tert-butoxy-ω-vinylbenzyl-polyglycidol. Their interfacial layer enriched in polyglycidol allowed the covalent immobilization of antigens complementary to antibodies against *Helicobacter pylori*, the proteins that are present in the blood of infected patients. The polyglycidol-rich interfacial layer eliminated any adventitious protein adsorption, allowing only attachment of antibodies against *Helicobacter pylori* by antigen–antibody interactions. Since both microspheres and antibodies are electrically charged, the attachment of antibodies affects the mobility of microspheres in an electric field.

## 4. Conclusions

Synthesis of polyglycidol with moderate molar mass (about 30,000) and controlled topology (linear, star-like, hyperbranched) does not pose a problem. Many methods were developed for synthesis of various copolymers containing units of polyglycidol, polyethers (mainly poly(ethylene oxide) and poly(propylene oxide)), polyesters (polylactide and poly(ε-caprolactone)), and polyolefins (styrene). These copolymers could be functionalized with alkene and alkyne moieties, sulfate, amine, azide, and carboxyl groups. They could be used for binding proteins and other biomolecules. Polyglycidols modified with hydrophobic segments self-assemble into polymeric micelles, polymeric liposomes, and other kinds of aggregates. When tethered to surfaces they modify the surface properties, making the surfaces hydrophilic and antifouling. Amphiphilic derivatives of polyglycidol are promising candidates for drug carriers and for contrast agents useful for various kinds of tissue imaging. However, in spite of significant achievements in this field, there are still several problems to be solved.

Many functionalization processes and synthetic routes of drug carriers are multistep and for large-scale pharmaceutical applications they are probably too expensive. Thus, a further search for simpler modification routes is still needed. Knowledge of relations between topology and chemical composition of polyglycidol-containing copolymers and their self-organization into polymeric micelles and other types of nanoparticles is insufficient and still requires further studies. In spite of numerous reports on protein–polyglycidol interactions, further investigation will be needed to give a detailed explanation of these interactions at a molecular level. The possibility of synthesizing high molar mass polyglycidol and polyglycidol-containing copolymers with biodegradable segments; *i.e.*, copolymers with mechanical properties appropriate for use as temporary implants replacing tendons and other elastic tissue, has been insufficiently explored. The field is still quite far from being fully explored.

## Figures and Tables

**Table 1:** Drug carriers based on polyglycidol and polyglycidol derivatives.

Encapsulator bioactive compound	Carrier	Method of encapsulation	Reference
Proteins			
BSA	Polylactide-hyperbranched polyglycidol nanoparticles	Nanoprecipitation	[68,69]
Insulin	β-cyclodextrin labeled polyglycidol nanoparticles	Coprecipitation	[70]
Insulin	β-cyclodextrin labeled polyglycidol hydrogel	Gelation	[71]
Asparaginase	Nanogels from dendritic polyglycidols with azide and -*p*-propargyloxy-benzacetale	Inverse nanoprecipitation	[72]
transglutaminase 1	Poly(N-isopropylacrylamide)-polyglycerol-based nanogels	Nanogel swelling in protein solution	[73]
Nucleic acids			
DNA	Quarternized a poly(glycidol-*co*-ethylene oxide)	Nanoprecipitation	[74]
pDNA	Quarternized branched poly(glycidol-*co*-2,3-epoxypropyldiethyl-amine) and DNA polyplexes	Incubation of polymer and nucleic acid solution	[75]
siRNA	Polyplexes of corona aminated hyperbranched polyglycidol and siRNA	Incubation of polymer and nucleic acid solution	[76]
siRNA	Polyplexes of hyperbranched polyglycidol modified with glycine and siRNA	Incubation of polymer and nucleic acid solution	[77]
siRNA	Polyplexes of amphiphilic copolymer containing hyperbranched polyglycidol end-capped with amines and siRNA	Incubation of polymer and nucleic acid solution	[78]
pDNA	Polyplexes of adamantane-modified hyperbranched polyglycerol end-capped with cationic β-cyclodextrin and pDNA	Incubation of polymer and nucleic acid solution	[79]
pDNA	Polyplexes of polyglycidol-pluronic-poly-glycidol with 2-(N,N-dimethylaminomethyl)-5-aminomethyl phenylboronic acid groups and pDNA	Incubation of polymer and nucleic acid solution	[80]
pDNA	Polyplexes of poly(ethylene oxide)-(branched polyglycidol) with endgroups capped with tris(2-aminoethyl)amine)	Incubation of polymer and nucleic acid solution	[81]
pDNA	nanodiamond particles with immobilized polyglycidol modified with grafted poly(arg-*co*-lys-*co*-his)	Incubation of nanocarrier and nucleic acid solution	[82]
pDNA and doxorubicin hydrochloride	Vesicles composed of cyclodextrin labeled with branched polyglycidol end-capped with tris(2-aminoethyl)amine) and bearing aliphatic chains	Nanoprecipitation	[83]
DNA Lamin A/C) and 5′-CTGGACTTCCAGAAGAACATT-3^−^	Polyplexes of hyperbranched polyglycidol modified by addition of oligoamines via the photo-cleavable linkages	Incubation of polymer and nucleic acid solution	[84]
Anticancer drugs			
Doxorubicin	Hyperbranched polyglycidol	Covalent immobilization via the enzymatically cleavable linkage	[85]
Doxorubicin	Hyperbranched polyglycidol with grafted poly(ethylene oxide)	Covalent immobilization via the enzymatically cleavable linkage	[86]
Doxorubicin	Poly(ethylene oxide)-[hyperbranched polyglycidol]	Covalent immobilization via pH sensitive hydrazone linkage	[87]
Doxorubicin	Hyperbranched polyglycidol with grafted poly(ethylene oxide) and targeting antibodies agains epidermal growth factor	Covalent immobilization via pH sensitive hydrazone linkage	[88]
Doxorubicin, indodicarbocyanine dye	Hyperbranched polyglycidol	Covalent immobilization of drug and dye via cleavable linkage. Monitoring of drug release by fluorescence	[89]
Doxorubicin	Nanodiamond particles with grafted hyperbranched polyglycidol end-capped with RGD tripeptide	Covalent immobilization via pH sensitive hydrazone linkage	[90]
Cisplatin	Hyperbranched polyglycidol modified with succinic anhydride	Covalent immobilization via carboxyl groups	[91]
Cisplatin	Nanodiamond particles with grafted hyperbranched polyglycidol end-capped with RGD tripeptide and COOH groups	Covalent immobilization via carboxyl groups	[92]
Methotrexate	Fe_3_O_4_ nanoparticles with immobilized hyperbranched polyglycidol	Covalent immobilization via pH sensitive hydrazone linkage	[93]
Paclitaxel	Hyperbranched polyglycidol with sulfate and amine end groups	Covalent immobilization via cleavable ester linkage	[94]
Paclitaxel	Hyperbranched polyglycidol with attached poly(ethylene oxide) and alkyl chains	Solubilization of hydrophobic drug	[95,96]
Paclitaxel	Polymeric micelles of hyperbranched polyglycidol labeled with β-cyclodextrin	Nanoprecipitation	[97]
Docetaxel	Hyperbranched polyglycidol with attached poly(ethylene oxide) and alkyl chains	Solubilization of hydrophobic drug	[96]
Docetaxel	Hyperbranched polyglycidol with attached alkyl chains	Nanoprecipitation	[98]
Sagopilone	Hyperbranched polyglycidol with attached alkyl chains	Entrapment during micellization	[99]
Miscellaneous			
Nimodipine	Hyperbranched polyglycidol with biphenyl groups in the core	Solubilization of hydrophobic drug	[100]
Quercetin	Polylactide-(hyperbranched polyglycidol)	Nanoprecipitation	[101]
Endomorphins	Hyperbrached polyglycidol modified by addition of poly(lactide-*co*-glycolide) chains	Water-in oil-in water double emulsification	[102]
